# Guiding light: new European guidelines on CLAD prevention and treatment

**DOI:** 10.3389/ti.2026.17855

**Published:** 2026-09-30

**Authors:** Kieran Halloran

**Affiliations:** Department of Medicine, University of Alberta, Edmonton, AB, Canada

**Keywords:** chronic lung allograft dysfunction, clinical practice guideline, clinical trial, lung transplantation, prevention

Chronic lung allograft dysfunction (CLAD) is a challenging clinical entity in lung transplantation. It is common, deadly, difficult to prevent, and almost entirely unresponsive to therapy. As the number one killer of lung transplant patients after the first year [1], it also limits lung transplant as a therapy for advanced lung disease both by reducing its cost-effectiveness and requiring stringent selection criteria to minimize its risks. It was described over four decades ago now after the first lung transplants in the 1980s (then referred to collectively as bronchiolitis obliterans syndrome or BOS) [2], but despite significant advances in the time since in understanding its pathology, clinical course, risk factors, outcome associations, physiologic and radiographic subtypes, and finally the development of a consensus definition [3], the lung transplant community has had only limited success in evolving meaningful strategies to address CLAD.

In recent years though, there have been important developments. The first successful multicenter randomized clinical trial (RCT) of CLAD prevention was recently completed by a consortium of Scandinavian countries (ScanCLAD), demonstrating that tacrolimus is superior to cyclosporine in preventing CLAD [4]. Although only a minority of centers were still using cyclosporine as *de novo* immunosuppression after transplant [5], this new evidence can and should change practice there. It also informs clinicians when making decisions about whether to rotate patients from tacrolimus to cyclosporine and for what indications it is worth the trade-off. The successful completion of a multicenter clinical trial studying the role of anti-fibrotic therapy in patients with the BOS subtype of CLAD–specifically, that it showed no benefit–has also helped move the needle on a perennial question in what is understood to be a fibrotic airways process [6]. A single-center RCT in Vienna has also now shown that extracorporeal photopheresis (ECP) may prevent CLAD, in addition to potentially lowering rates of rejection and infection as well [7]. These works build on a previous successful Belgian clinical trial showing that azithromycin prophylaxis can reduce CLAD risk [8].

Despite these advances though, the evidence for CLAD therapeutics remains mixed or entirely lacking, and this can result in heterogeneity in the clinical approach [9, 10]. CLAD is a universally feared complication, particularly in its rapidly progressive forms, and clinicians faced with an ailing patient and armed with limited evidence do not tend to stand by. The interventions clinicians impose are well-intentioned and may in some cases be beneficial but can set the stage for patient- and system-level harms, such as adverse drug effects, infectious or malignant complications of immunosuppression, and resource utilization without clear benefit. It is these contexts where clinical practice guidelines can make a difference by evaluating the evidence, providing grounded recommendations, and highlighting the important gaps that need to be addressed.

Enter the European Society for Organ Transplantation (ESOT) CLAD guidelines, reported in this issue of *Transplant International* [11]. A panel of noted European lung transplant experts spanning 8 countries has produced a document that at least in part fills this role. The group conducted a comprehensive literature search resulting in 8 recommendations addressing various CLAD prevention and treatment subjects, including macrolide therapy, maintenance immunosuppressive choices, extracorporeal photopheresis, and anti-rejection therapies, among others. The choice of what topics to address is strategic, as these reflect some of the most widely used and widely available therapies for CLAD, some of which have significant risk such as anti-thymocyte globulin and alemtuzumab. It should be noted that this is not the first guideline on CLAD management. A 2014 joint statement of the International Society for Heart and Lung Transplantation (ISHLT), European Respiratory Society, and American Thoracic Society summarized potential treatment strategies for CLAD (then BOS), in addition to diagnostic recommendations, though there was limited focus on prevention. This document also predated the 2019 definition consensus statement and particularly given the evolution of the evidence-base in the interim, a new guideline statement 12 years later is logical and much needed.

Due to limitations imposed by the ESOT guideline format, which restricts these documents to 8 PICO questions and associated recommendations, the authors acknowledge that they could not delve comprehensively into other important topics which are relevant to CLAD prevention and treatment. These include the role of induction therapy, palliative care strategies, retransplantation, anti-reflux surgery, corticosteroid therapy, and others. While understandable, these are regrettable omissions which render the document non-definitive and mean the community must await either a new iteration of this document or another guideline from another organization. Induction therapy and retransplantation in particular are important aspects of transplant care on which guidance is needed, particularly given practice is heterogenous [12]. There have been several clinical trials on induction therapy in lung transplantation, but none have demonstrated an effect, and these drugs can have risks for patients with frequent infections [13, 14]. Meanwhile retransplantation is a critical therapeutic resource for advanced CLAD in an otherwise well patient but one which is difficult to balance against first-time transplants in donor resource-constrained settings. While it is addressed by the ISHLT candidate selection guidelines, this document would seem an ideal place to formally comment on its use.

Considered on its own merits though, this author group has produced a meaningful evidence-based document with measured clinical guidance that will be helpful for smaller/newer lung transplant programs, in training lung transplant clinicians, and in helping existing programs re-evaluate their current approaches. Most importantly, it sheds light on the area most in need of study: CLAD therapeutics. It is this author’s hope that with the evolution of the evidence base in recent years, armed with a standard definition and consensus endpoints [15], and with the current trials underway, we are entering an era in which the next iteration of a CLAD management document contains evidence for therapies which are not only effective in preventing CLAD, but in treating patients already affected by it.

## Data Availability

The original contributions presented in the study are included in the article/supplementary material, further inquiries can be directed to the corresponding author.
